# Inhibitory Effects of Constituents from *Euphorbia lunulata* on Differentiation of 3T3-L1 Cells and Nitric Oxide Production in RAW264.7 Cells

**DOI:** 10.3390/molecules16108305

**Published:** 2011-09-29

**Authors:** Zhi-Gang Yang, Liu-Nan Jia, Yan Shen, Atsuko Ohmura, Susumu Kitanaka

**Affiliations:** 1 School of Pharmacy, Nihon University, 7-7-1 Narashinodai, Funabashi, Chiba 274-8555, Japan; Email: yang_zhg@hotmail.com (Z.-G.Y.); deathmetallica82@hotmail.com (L.-N.J.); 2 College of Bioresource Sciences, Nihon University, 1866 Kameino, Fujisawa, Kanagawa 252-8510, Japan; Email: shen.yan@nihon-u.ac.jp (Y.S.); 3 Saitama Prefectural Pharmaceutical Affairs Divison, 3-15-1 Takasago, Saitama, Saitama 330-9301, Japan; Email: omura.atsuko@pref.saitama.lg.jp (O.A.)

**Keywords:** *Euphorbia lunulata* Bge., flavonol galactopyranoside gallate, phenolic constituents, adipocyte, macrophage

## Abstract

A new flavonol galactopyranoside, myricetin 3-*O*-(2'',3''-digalloyl)-β-D-galactopyranoide (**1**), and 23 known constituents, including myricetin 3-*O*-(2''-galloyl)-β-D-galactopyranoide (**2**), myricitrin (**3**), myricetin (**4**), quercetin 3-*O*-(2'', 3''-digalloyl)-β-D-galactopyranoide (**5**), quercetin 3-*O*-(2''-galloyl)-β-D-galactopyranoide (**6**), hyperin (**7**), isoquercetrin (**8**), quercetin (**9**), kaempferol (**10**), apigenin (**11**), luteolin (**12**), 3-*O*-methylquercetin (**13**), 5,7,2',5'-tetrahydroxyflavone (**14**), 1,3,4,6-*tetra*-*O*-galloyl-β-D-glucose (**15**), 1,2,6-*tri*-*O*-galloyl-β-D-glucose (**16**), 1,3,6-*tri*-*O*-galloyl-β-D-glucose (**17**), gallic acid (**18**), protocatechuic acid (**19**), 3,4,5-trimethoxybenzoic acid (**20**), 2,6-dihydroxyacetophenone (**21**), 3,3'-*di*-*O*-methylellagic acid (**22**), ellagic acid (**23**) and esculetin (**24**) were isolated from *Euphorbia lunulata* Bge. Their structures were determined by spectroscopic analysis. Isolated hydrolysable tannins, flavonoids, and flavonol galactopyranoside gallates showed significant inhibition of the differentiation of 3T3-L1 preadipocytes and triglyceride accumulation in maturing adipocytes, and nitric oxide production in RAW 264.7 cells.

## 1. Introduction

*Euphorbia lunulata* Bge. (Euphorbiaceae) is a perennial herbaceous plant that has long been used in China as a traditional crude drug for the treatment of chronic bronchitis. Previous investigations of *E. lunulata* Bge. have yielded some flavonoids and coumarins that have been reported to have anticancer, antioxidant, insulin and interleukin-10 (IL-10) mimicking activities [1,2,3,4,5].

It is well known that obesity is often accompanied by hyperglycemia, hypertension, and hyperlipidemia, which are together known as metabolic syndrome. Obesity is the result of an increase in the number and size of adipocytes, in which adipocytes accumulate a large amount of lipids and become enlarged. Cultured 3T3-L1 adipocytes show many properties similar to those of normal adipocytes, lipids are accumulated during the differentiation and maturing process, the cytosolic enzyme glycerol-3-phosphate dehydrogenase (GPDH) appears to have an important role in the conversion of glycerol into triglyceride (TG), and the level of activity of GPDH increases during the conversion of 3T3 cells. Recent studies indicate that obesity is associated with low-grade chronic inflammation of adipose tissues, and the obese adipose tissue is characterized by increased infiltration of macrophages. In a coculture system of 3T3-L1 adipocytes and RAW264 macrophages, marked increases in secretion levels of inflammatory mediators such as TNF-α, MCP-1, and nitric oxide (NO) were observed [6,7,8]. NO mediates diverse functions by acting on various cells through interactions with different molecular targets, and excessive NO production is involved in various types of inflammation [9]. Therefore, as a part of our continuing investigation of anti-adipogenesis and anti-inflammatory agents from natural sources [10], the chemical composition of the whole plant of *E. lunulata* Bge. has been examined.

**Figure 1:**
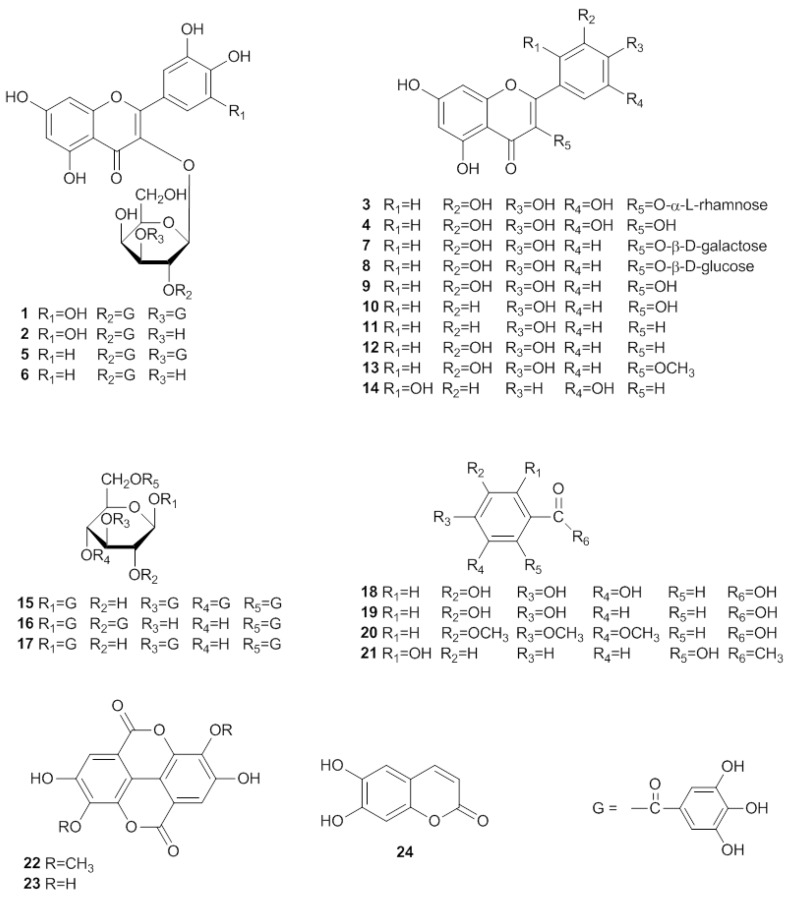
Structures of compounds **1–24**.

In the present study, the 70% acetone extract of the whole plant of *E. lunulata* Bge. and the ethyl acetate soluble portion of the extract were observed to inhibit the accumulation of TG in 3T3-L1 cells, and inhibit NO production in lipopolysaccharide (LPS) and interferon-γ (IFN-γ) activated macrophages. Furthermore, an attempt to identify the bioactive compounds present in this plant led to the isolation of a new flavonol galactopyranoside gallate, myricetin 3-*O*-(2'',3''-digalloyl)-β-D-galactopyranoide (**1**), and 23 known constituents, including myricetin 3-*O*-(2''-galloyl)-β-D-galactopyranoide (**2**) [11], myricitrin (**3**) [12], myricetin (**4**) [13], quercetin 3-*O*-(2'',3''-digalloyl)-β-D-galactopyranoide (**5**) [2], quercetin 3-*O*-(2''-galloyl)-*β*-D-galactopyranoide (**6**) [14], hyperin (**7**) [15], isoquercetrin (**8**) [16], quercetin (**9**) [17], kaempferol (**10**) [18], apigenin (**11**) [19], luteolin (**12**) [19], 3-*O*-methylquercetin (**13**) [20], 5,7,2',5'-tetrahydroxyflavone (**14**) [21], 1,3,4,6-*tetra*-*O*-galloyl-β-D-glucose (**15**) [22], 1,2,6-*tri*-*O*-galloyl-β-D-glucose (**16**) [23], 1,3,6-*tri*-*O*-galloyl-β-D-glucose (**17**) [24], gallic acid (**18**) [25], protocatechuic acid (**19**) [26], 3,4,5-trimethoxybenzoic acid (**20**) [27], 2,6-dihydroxyacetophenone(**21**) [28], 3,3'-*di*-*O*-methylellagic acid (**22**) [29], ellagic acid (**23**) [30] and esculetin (**24**) [31] (Figure 1). Among these compounds, **1–4**, **8**, **11–17**, and **19–22** were isolated from *E. lunulata* Bge. for the first time. We also describe their inhibitory activity towards the differentiation of 3T3-L1 preadipocytes and TG accumulation in maturing adipocytes, and their inhibition of NO production in RAW 264.7 cells in this contribution.

## 2. Results and Discussion

Whole plants of *Euphorbia lunulata* Bge. were extracted with 70% aqueous acetone, followed by evaporation of the solvent under reduced pressure from the combined extract to give the 70% acetone extract. The extract [TG and NO inhibitory activity: 50%, 89%, respectively (100 μg/mL)] was dissolved in water and successively partitioned to give a chloroform layer [68%, 71% (30 μg/mL)], ethyl acetate layer [70%, 76% (30 μg/mL)], *n*-butanol layer [41%, 23% (30 μg/mL)], and aqueous layer [32%, 8% (30 μg/mL)]. We have focused our efforts on investigating the chemical constituents of the ethyl acetate soluble fraction, which was separated by Diaion HP-20 column chromatography to yield eight fractions from which twenty three compounds were isolated. One compound was isolated from the chloroform layer.

**Figure 2:**
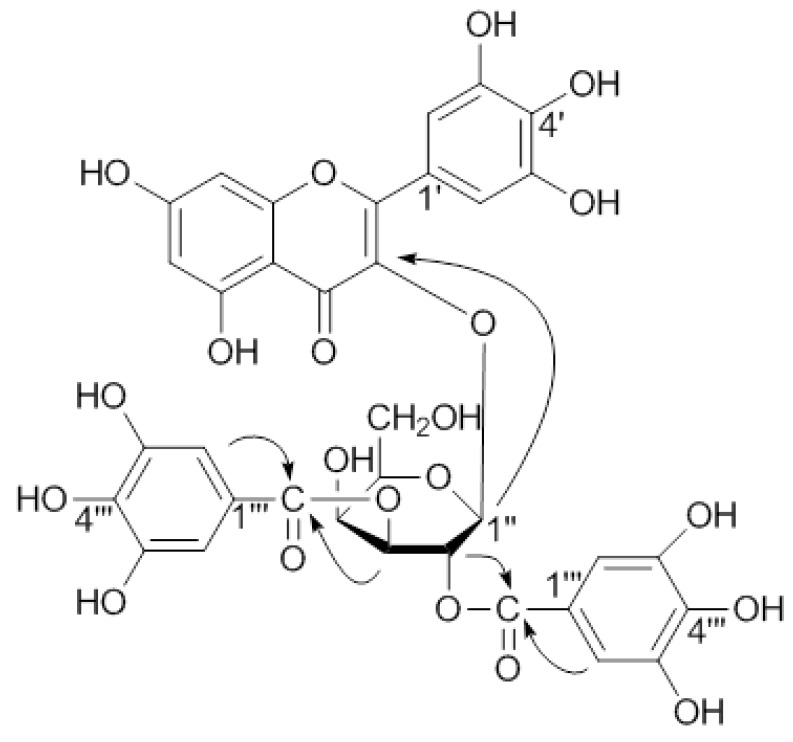
Key HMBC correlations of **1**.

Compound **1** was obtained as a yellow amorphous powder and gave positive Mg-HCl and FeCl_3_ tests. The molecular formula of **1** was established as C_35_H_28_O_21_ by HR-FAB-MS. The UV spectrum exhibited absorption maxima at 268 and 355 nm, suggesting the presence of aromatic rings in the molecule. The IR spectrum contained absorption bands for a hydroxyl group (3,396 cm^−1^), ester carbonyl (1,703 cm^−1^) and conjugated carbonyl (1,656 cm^−1^), and aromatic ring (1,608, 1,497 cm^−1^). The ^1^H-NMR spectrum of **1** indicated *meta*-coupled proton signals at δ 6.31 (1H, d, *J* = 2.1 Hz) and 6.14 (1H, d, *J* = 2.1 Hz), and an A_2_-type aromatic proton signal at δ 7.27 (2H, s). These data for the aromatic region matched those of myricetin 3-*O*-(2''-galloyl)-β-D-galactopyranoide (**2**) and myricetin (**4**), indicating the aglycone was most likely a myricetin (Table 1). Except for the aglycone signals, the ^1^H- and ^13^C-NMR spectra of **1** showed the presence of two galloyl groups [δ_H_ 7.03, 7.02, (each 2H, s); δ_C_ 166.4 (C=O), 166.2 (C=O), 145.1 (×4), 138.8, 138.7, 119.1, 118.7, 109.4 (×2), 109.3 (×2)], and a sugar of the anomeric proton signal at δ 5.94 (1H, d, *J* = 8.1 Hz). The sugar was found to be ^4^C_1_ galactopyranose with the help of ^1^H-^1^H COSY. These data coupled with the FAB-MS data indicated that **1** may be a digallate of myricetin galactopyranoside. Myricetin, gallic acid, and D-galactose were identified by hydrolysis of **1** (see Experimental). Furthermore, comparative analysis of the ^13^C-NMR data of the aglycone carbons in **1** with those of **4** showed an upfield shift of C-3 (*Δ*δ 1.6 ppm), a downfield shift of C-2 (*Δ*δ 9.7 ppm) and C-4 (*Δ*δ1.8 ppm), suggesting the location of the sugar was at C-3 [32]. This was further supported by HMBC correlations, the anomeric proton signal δ 5.94 (H-1'') showed long-range correlation with the carbon signal at δ 134.0 (C-3) (Figure 2). The locations of the two galloyl groups in **1** were confirmed to be at C-2'' and C-3'' of the galactosyl moiety by the remarkable downfield shifts of H-2'' (δ 5.81, dd, *J* = 10.2, 8.1 Hz), and H-3'' (δ 5.18, dd, *J* = 10.2, 3.0 Hz) signals, a finding which was further supported by HMBC correlations between the sugar protons at δ5.81 and δ5.18 with the carbon signals (δ 166.4, 166.2) of the two galloyl carbonyls (Figure 2). Based on the foregoing findings, **1** was determined to be myricetin 3-*O*-(2'',3''-digalloyl)-β-D-galactopyranoide.

**Table 1:** ^1^H- and ^13^C-NMR Spectral Data of compounds **1**, **2** and **4** [(300/75 MHz, in methanol-*d*_4_, TMS, δ (ppm) (*J* = Hz)].

Position	1		2		4	
	δ_H_	δ_C_	δ_H_	δ_C_	δ_H_	δ_C_
2		156.4		156.6		146.7
3		134.0		134.0		135.6
4		177.6		177.4		175.8
4a		104.6		104.7		103.3
5		161.7		161.7		161.1
6	6.14 (d 2.1)	98.5	6.13 (d 2.1)	98.5	6.17 (d 2.1)	98.1
7		164.5		164.4		164.2
8	6.31 (d 2.1)	93.3	6.30 (d 2.1)	93.3	6.36 (d 2.1)	93.2
8a		156.6		156.8		156.8
1'		120.6		120.8		121.8
2'	7.27 (s)	108.6	7.25 (s)	108.6	7.32 (s)	107.3
3'		144.9		144.9		145.4
4'		136.6		136.6		136.1
5'		144.9		144.9		145.4
6'	7.27 (s)	108.6	7.25 (s)	108.6	7.32 (s)	107.3
1''	5.94 (d 8.1)	100.1	5.77 (d 7.8)	100.2		
2''	5.81 (dd 10.2, 8.1)	74.8	5.43 (dd 9.9, 7.8)	73.5		
3''	5.18 (dd 10.2, 3.0)	70.7	3.84 (dd 9.9, 3.6)	72.4		
4''	4.28 (d 3.0)	66.9	3.95 (d 3.6)	69.5		
5''	3.75 (m)	76.3	3.61 (m)	76.4		
6''	3.71, 3.69 (m)	60.8	3.71, 3.69 (m)	61.0		
1'''		119.1, 118.7		120.4		
2''',6'''	7.03, 7.02 (s)	109.4, 109.3	7.12 (s)	109.5		
3''',5'''		145.1		145.0		
4'''		138.8, 138.7		138.5		
C=O		166.4, 166.2		166.7		

Compounds **2–24** were identified as known compounds by detailed comparisons of their spectroscopic data with that in the literature.

**Figure 3:**
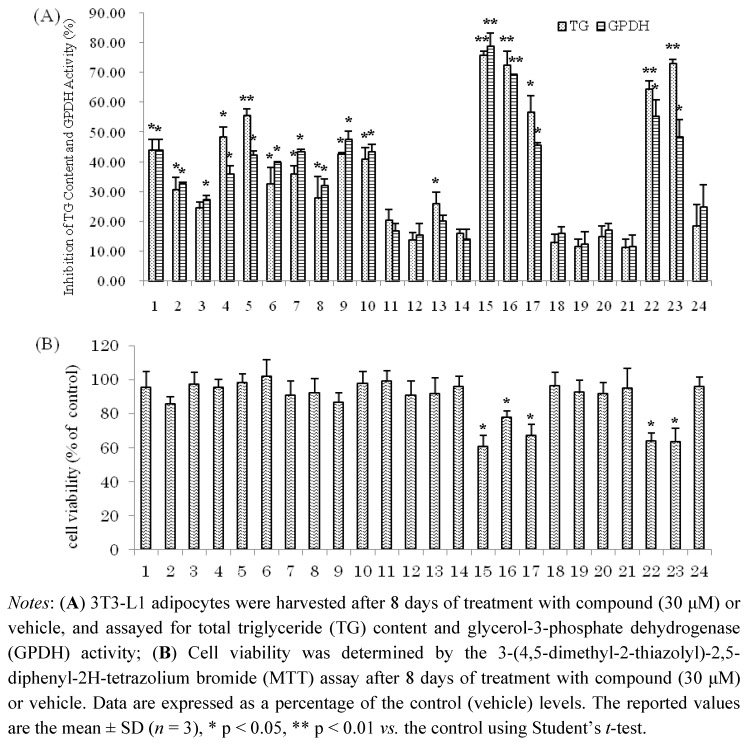
(**A**) Inhibition of TG Content and GPDH Activity by the isolated compounds in 3T3-L1 Adipocytes; (**B**) Cell viability of 3T3-L1 cells by MTT assay.

**Figure 4:**
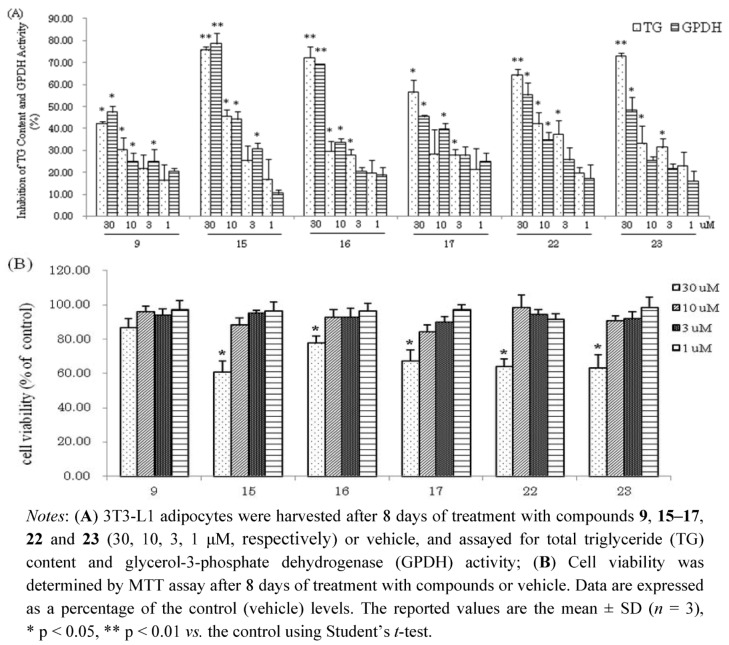
(**A**) Inhibition of TG Content and GPDH Activity by compounds **9**, **15**-**17**, **22** and **23** at low concentrations in 3T3-L1 Adipocytes; (**B**) Cell viability of 3T3-L1 cells by MTT assay.

Compounds **1–24** were next evaluated for their inhibitory effects on triglyceride accumulation and GPDH activity in 3T3-L1 cells at the concentration of 30 μM (Figure 3). The major component, quercetin (**9**, 0.038% yield), which has been reported to have inhibitory effects on triglyceride accumulation, was used as a positive control [10,33]. As shown in Figure 3, the hydrolysable tannins, **15–17**, **22** and **23** showed significant inhibition of adipogenesis in 3T3-L1 adipocytes, with TG inhibition values of 75.7%, 72.2%, 56.6%, 64.2% and 72.8%, respectively. The flavonol galactopyranoside gallates, **1**, **2**, **5** and **6** showed moderate TG inhibitory activities (43.7%, 30.5%, 55.5% and 32.5% inhibition, respectively). Comparing **1** with **2** and **5** with **6**, compounds **1**, **5** showed stronger TG and GPDH activity inhibition than **2** and **6**, respectively; these results suggest that the galloyl group may enhance their activities. The flavonols, **7**, **8**, **9**, **10** and **13** showed moderate TG inhibitory activities (35.8%, 27.7%, 42.4%, 40.8% and 25.9% inhibition, respectively), which were stronger than the flavones, **11**, **12** and **14** with TG inhibitions 20.3%, 13.7%, and 15.9%, respectively. The phenylpropanoids **18–21** and coumarin **24** showed weak inhibitory effects on TG and GPDH activity. Furthermore, effects of isolated compounds on cell viability of 3T3-L1 cells were measured by MTT assay. As shown in Figure 3, the isolated compounds of **15–17**, **22** and **23** had effects on viability of 3T3-L1 at concentration of 30 μM. However, these compounds at low concentrations (10, 3, 1 μM, respectively) suppressed the differentiation of 3T3-L1 preadipocytes and TG accumulation in maturing adipocytes without exerting cytotoxicity (Figure 4). The hydrolysable tannin, **15** and **22** showed significant inhibitions of TG content and GPDH activity at the concentration of 10 μM, **15** with values of 45.6% and 44.2%, **22** with values of 42.0% and 34.7%, respectively.

**Figure 5:**
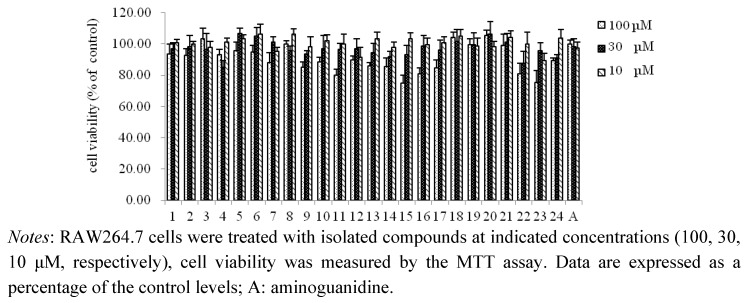
Effects of isolated compounds on cell viability of RAW264.7 cells by MTT assay.

Compounds **1–24** also examined with respect to their inhibition of NO production stimulated by LPS and IFN-*γ* in RAW 264.7 cells (Table 2). In the assay, aminoguanidine, which has been reported to have inhibitory effects on NO production in LPS activated RAW 264.7 macrophages by down-regulation of inducible nitric oxide synthase (iNOS), was used as a positive control (IC_50_ 17.5 μM) [34]. As shown in Table 2, in general, all compounds except **3**, **7**, **8**, **18**, **20**, **21** and **24** showed NO production inhibitory activities with IC_50_ values lower than 100 μM. Furthermore, the hydrolysable tannins, **15** and **17**, showed slightly stronger NO production inhibitory effects than aminoguanidine, with IC_50_ values of 14.2 and 16.9 μM, respectively; compounds **16**, **22** and **23** showed moderate NO inhibitory activity, with IC_50_ values of 21.7, 18.5 and 26.1 μM, respectively. These results showed that the hydrolysable tannins were the significantly active compounds in *E. lunulata* Bge. In addition, the free flavonoids, **4**, **9**, **10**, **11**, **12**, **13** and **14**, showed strong or moderate NO inhibitory activity, with IC_50_ values of 37.2, 20.6, 21.5, 17.9, 23.8, 42.6 and 18.5μM, respectively. The flavonol glycosides, **3**, **7** and **8**, showed no inhibition. However, the flavonol galactopyranoside gallates, **1**, **2**, **5** and **6**, showed little or moderate NO inhibitory activity, with IC_50_ values of 62.3, 48.6, 56.8 and 43.2 μM, respectively. In contrast, the descending orders of the NO inhibitory activities were: **4** > **2** > **1** > **3** (myricetin derivatives), and **9** > **6** > **5** > **7**, **8** (quercetin derivatives). These results showed that the free flavonoids exerted stronger activities than the corresponding glycosides, and the conjugation of gallic acid may have enhanced the activities of the flavonoid glycosides, even though gallic acid (**18**) itself showed no inhibition. Furthermore, effects of isolated compounds on cell viability of RAW264.7 cells were measured by MTT assay. As shown in Figure 5, the isolated compounds had no effects on viability of RAW264.7 at concentration of IC_50_ values.

**Table 2:** Inhibition by isolated compounds of NO production stimulated by LPS and IFN-γ in RAW 264.7 cells.

Compound	IC_50_ (μM)	Compound	IC_50_ (μM)
**1**	62.3	**13**	42.6
**2**	48.6	**14**	18.5
**3**	>100	**15**	14.2
**4**	37.2	**16**	21.7
**5**	56.8	**17**	16.9
**6**	43.2	**18**	>100
**7**	>100	**19**	19.8
**8**	>100	**20**	>100
**9**	20.6	**21**	>100
**10**	21.5	**22**	18.5
**11**	17.9	**23**	26.1
**12**	23.8	**24**	>100
Aminoguanidine	17.5		

## 3. Experimental

### 3.1. General

The UV spectrum was obtained in MeOH on a Shimadzu UV-160 spectrophotometer, and the IR spectrum was recorded on a JASCO FT/IR 300E spectrophotometer. Optical rotations were measured in MeOH on a JASCO P-1020 polarimeter. The NMR spectra were recorded on a Varian Mercury 300 spectrometer, with TMS as an internal standard. The MS were obtained on a JEOL GCmate mass spectrometer. Column chromatography was carried out with Diaion HP-20 (Mitsubishi Chemical Co.), Sephadex LH-20 (Pharmacia), MCI Gel CHP20P (Mitsubishi Chemical Co.) and Chromatorex ODS (Fuji Silysia Chemical Ltd.). Thin-layer chromatography (TLC) was performed on Merck TLC plates (0.25 mm thickness), with compounds visualized by spraying with 5% (v/v) H_2_SO_4_ in ethanol solution and then heating on a hot plate. HPLC was performed on a JASCO PU-2089 apparatus equipped with JASCO UV-2075 and Shodex OR-2. Senshu Pak Silica 60-5(10 × 150 mm i.d.), YMC-Pack Pro C18 (10 × 150 mm i.d.), YMC-Pack ODS Al (10 × 150 mm i.d.) and Cosmosil 5C18 PAQ (10 × 150 mm i.d.) was used for preparative purposes. A CAPCELL Pak NH_2_ column (4.6 × 250 mm i.d.) was used for the detection of D-galactose.

### 3.2. Plant Material

Whole plants of *E. lunulata* Bge. were collected in Hebei Province, China, in October 2006 and were identified by Professor Naili Wang (Shenyang Pharmaceutical University, China). Voucher specimens have been deposited at the Laboratory of Pharmacognosy, School of Pharmacy, Nihon University.

### 3.3. Extraction and Isolation

The whole plants of *E. lunulata* Bge. (4.75 kg) were extracted twice with 70% acetone and concentrated to give the extract. This extract (721 g) was dissolved and suspended in water (3 L) and partitioned into a chloroform layer (81 g), ethyl acetate layer (50 g), *n*-butanol layer (81 g) and water layer (490 g). The ethyl acetate layer was subjected to Diaion HP-20 column chromatography and eluted with CH_3_OH-H_2_O to afford eight fractions. Fractions 1 and 2 were purified using Sephadex LH-20 column chromatography and eluted with CH_3_OH-H_2_O to give **3** (856 mg) and **16** (88 mg), respectively. Fraction 3 was purified using Sephadex LH-20 and ODS column chromatography, and reverse-phase HPLC eluted with CH_3_OH-H_2_O to give **1** (9 mg), **2** (13 mg), **5** (15 mg), **6** (607 mg), **7** (200 mg), **9** (68 mg), **15** (39 mg), **16** (5 mg) and **17** (7 mg). Fraction 4 was purified using CHP20P column chromatography and reverse-phase HPLC eluted with CH_3_OH-H_2_O to give **3 **(10 mg), **4** (8 mg), **8** (11 mg) and **20** (26 mg). Fraction 5 was purified using CHP20P column chromatography and reverse-phase HPLC eluted with CH_3_OH-H_2_O to give **9** (1,800 mg), **12** (18 mg) and **14** (6 mg). Fraction 6 was purified using CHP20P column chromatography and reverse-phase HPLC eluted with CH_3_OH-H_2_O to give **10** (9 mg), **11** (10 mg), **13** (6 mg), **21** (5 mg), **22** (125 mg) and **23** (68 mg).The chloroform layer was subjected to silica gel column chromatography and reverse-phase HPLC to give **24** (25 mg).

*Myricetin 3-O-(2'',3''-digalloyl)-β-*D-*galactopyranoide* (**1**). Yellow amorphous powder; Negative HR-FAB-MS *m/z*: 783.1040 (Calcd for C_35_H_27_O_21_, 783.1043); [α]_D_^25^ −18.9° (c = 0.10, MeOH). UV (MeOH) λ_max_ nm (log ε): 210 (4.62), 268 (4.27), 355 (3.96); IR (KBr) ν_max_ cm^−1^: 3,396, 1,703, 1,656, 1,608, 1,497, 1,451, 1,352, 1,300, 1,272, 1,231, 1,075, 1,036, 942, 876, 760; ^1^H- and ^13^C-NMR data, see Table 1.

### 3.4. Acid Hydrolysis of 1 and Identification of Sugar

Compound **1** (5 mg) was dissolved in 10% H_2_SO_4_ and heated at 85 °C for 3 h. After cooling, the reaction mixture was neutralized by passage though an Amberlite IRA-93ZU (Organo) column and then partitioned between ethyl acetate and water. The ethyl acetate layer was concentrated and the concentrate was passed through an ODS column, and then successively eluted with 20% MeOH and 50% MeOH. Gallic acid was recovered from the 20% MeOH fraction, and myricetin from the 50% fraction by direct comparison with authentic samples. Silica gel TLC of the water layer in BuOH:Me_2_CO:H_2_O (4:5:1) showed the sugar was most likely a galactose (R_f_ 0.22) by direct comparison with authentic sample. The water layer was then analyzed by HPLC under the following conditions: column, CAPCELL Pak NH_2_ (4.6 × 250 mm i.d.); solvent, CH_3_CN:H_2_O = 75:25; flow rate 1.0 mL/min; detector, Shodex OR-2. D-galactose present in the water layer was identified by comparing its retention time and polarity with those of an authentic sample; tR (min):16.8 min (D-galactose, positive polarity).

### 3.5. Triglyceride (TG) Content and Glycerol-3-phosphate Dehydrogenase (GPDH) Activity in 3T3-L1 Cells *[10,35]*

3T3-L1 preadipocytes (American Type Culture Collection, Manassas, VA, USA) were subcultured in Dulbecco’s Modified Eagle’s Medium (DMEM) containing 10% newborn calf serum (Gibco) at 37 °C under a humidified 5% CO_2_ atmosphere. Briefly, 2 days after reaching confluence (day 0), the 3T3-L1 preadipocytes were induced by switching the differentiation medium to DMEM containing 10% fetal bovine serum (FBS) (SAFC Biosciences), 500 μM 3-isobutyl-1-methylxanthine (Sigma), 1 μM dexamethasone (Sigma), and 10 μg/mL insulin (Sigma) for three days. The medium was then replaced with DMEM containing 10% FBS and 5 μg/mL insulin, and was changed every 2 or 3 days.

The 3T3-L1 preadipocytes were seeded at 1.0 × 10^5^ cells/mL onto 24-well plates (Sumitomo Bakelite, MS-80240, Tokyo) and incubated at 37 °C. A test sample was added to the medium on day 0, and added at the time of every medium change during the 8 days of incubation. After removing the medium, the cells were washed twice with 500 μL of PBS. The cells were collected in 500 μL of cold sonication buffer (pH 7.5 25 mM Tris buffer containing 1 mM EDTA) and sonicated in ice-cold water. After centrifugation, the cell lysate was used to measure the TG content with LabAssay^TM^ Triglyceride (WAKO Pure Chemical Industries Ltd.), GPDH activity with a GPDH Assay Kit (TaKaRa Bio Inc.), and DNA quantity with a DNA Quantity Kit (Primary Cell Co., Ltd.), according to the manufacturer's protocol. Inhibition of TG and GPDH activity was calculated using the following formula: Inhibition (%) = [(*Cn* − *S*)/*Cn*] × 100, where *S* is TG amount or GPDH activity when cells incubated with sample and divided by the amount of DNA for each well; *Cn* is TG amount or GPDH activity when cells incubated with DMSO (control) were divided by the amount of DNA for each well). Cell viability was confirmed by microscopic observation, and was measured using the 3-(4,5-dimethyl-2-thiazolyl)-2,5-diphenyl-2*H*-tetrazolium bromide (MTT) assay method.

### 3.6. NO Production in Activated Macrophage-Like Cell Line, RAW 264.7 *[10,36]*

The macrophage-like cell line, RAW 264.7, was obtained from American Type Culture Collection. The cells were cultured in Ham’s F12 medium with 10% FBS (SAFC Biosciences) at 37 °C under a humidified 5% CO2 atmosphere. The RAW264.7 cells were seeded at 1.2 × 106 cells/mL onto 96-well plates (Sumitomo Bakelite, MS-8096R, Tokyo) and then incubated at 37 °C for 2 h. A test sample was then added to the culture simultaneously with both Escherichia coli LPS (100 ng/mL) and recombinant mouse IFN-γ (0.33 ng/mL), and the cells were incubated at 37 °C, usually for 16 h. The amount of nitrite in culture supernatants was measured using the Griess assay. Cytotoxicity was measured using the MTT assay method.

### 3.7. Statistical Analysis

The values represent mean ± S.D. of at least three experiments. Statistical significant differences were evaluated by the one-way ANOVA followed by the Student’s *t*-test for paired populations, and a P value of 0.05 or less among the means were considered significant.

## 4. Conclusions

Obesity is associated with low-grade chronic inflammation of adipose tissues, and obese adipose tissue is characterized by increased infiltration of macrophages. The whole plant extract of *E. lunulata Bge*. showed inhibitory effects on the differentiation of 3T3-L1 cells and NO production in RAW264.7 cells. Bioassay-directed investigation of active constituents, led to the identification of a new flavonol galactopyranoside gallate, myricetin 3-*O*-(2'',3''-digalloyl)-β-D-galactopyranoide (**1**) and 23 known phenolic constituents. The isolated compounds, especially hydrolysable tannins, flavonoids, and flavonol galactopyranoside gallates, showed significant inhibition towards the differentiation of 3T3-L1 adipocytes and nitric oxide production in RAW 264.7 cells. These studies suggest that the extract of *E. lunulata Bge*. and the isolated compounds might be a source of anti-obesity and anti-inflammatory agents to improve the symptoms of metabolic syndrome.
